# Silent infarction in sickle cell disease is associated with brain volume loss in excess of infarct volume

**DOI:** 10.3389/fneur.2023.1112865

**Published:** 2023-03-31

**Authors:** R. Sky Jones, Manus J. Donahue, L. Taylor Davis, Sumit Pruthi, Spencer L. Waddle, Chelsea Custer, Niral J. Patel, Michael R. DeBaun, Adetola A. Kassim, Mark Rodeghier, Lori C. Jordan

**Affiliations:** ^1^Division of Pediatric Neurology, Department of Pediatrics, Vanderbilt University Medical Center, Nashville, TN, United States; ^2^Department of Radiology, Vanderbilt University Medical Center, Nashville, TN, United States; ^3^Department of Neurology, Vanderbilt University Medical Center, Nashville, TN, United States; ^4^Department of Psychiatry, Vanderbilt University Medical Center, Nashville, TN, United States; ^5^Vanderbilt-Meharry Center of Excellence in Sickle Cell Disease, Nashville, TN, United States; ^6^Division of Hematology and Oncology, Department of Medicine, Vanderbilt University Medical Center, Nashville, TN, United States; ^7^Rodeghier Consulting, Chicago, IL, United States

**Keywords:** sickle cell, infarction, brain volume, cerebral blood flow, MRI, silent cerebral infarct

## Abstract

**Introduction:**

Sickle cell disease (SCD) increases cerebral infarct risk, but reported effects on brain volume have varied. More detailed information using larger cohorts and contemporary methods could motivate the use of longitudinal brain volume assessment in SCD as an automated marker of disease stability or future progression. The purpose of this study was to rigorously evaluate whether children and young adults with SCD have reduced gray matter volume (GMV) and white matter volume (WMV) compared to healthy controls using high-resolution MRI. We tested the hypotheses that (i) elevated CBF, a marker of cerebral hemodynamic compensation in SCD, is associated with global and regional brain atrophy, and (ii) silent cerebral infarct burden is associated with brain atrophy in excess of infarct volume.

**Methods:**

Healthy controls (*n* = 49) and SCD participants without overt stroke (*n* = 88) aged 7–32 years completed 3 T brain MRI; pseudocontinuous arterial spin labeling measured CBF. Multivariable linear regressions assessed associations of independent variables with GMV, WMV, and volumes of cortical/subcortical regions.

**Results:**

Reduced hemoglobin was associated with reductions in both GMV (*p* = 0.032) and WMV (*p* = 0.005); reduced arterial oxygen content (CaO_2_) was also associated with reductions in GMV (*p* = 0.035) and WMV (*p* = 0.006). Elevated gray matter CBF was associated with reduced WMV (*p* = 0.018). Infarct burden was associated with reductions in WMV 30-fold greater than the infarct volume itself (*p* = 0.005). Increased GM CBF correlated with volumetric reductions of the insula and left and right caudate nuclei (*p* = 0.017, 0.017, 0.036, respectively). Infarct burden was associated with reduced left and right nucleus accumbens, right thalamus, and anterior corpus callosum volumes (*p* = 0.002, 0.002, 0.009, 0.002, respectively).

**Discussion:**

We demonstrate that anemia and decreased CaO2 are associated with reductions in GMV and WMV in SCD. Increased CBF and infarct burden were also associated with reduced volume in subcortical structures. Global WMV deficits associated with infarct burden far exceed infarct volume itself. Hemodynamic compensation via increased cerebral blood flow in SCD seems inadequate to prevent brain volume loss. Our work highlights that silent cerebral infarcts are just a portion of the brain injury that occurs in SCD; brain volume is another potential biomarker of brain injury in SCD.

## Introduction

Sickle cell disease (SCD) is a chronic hemolytic anemia (1) Silent cerebral infarcts (SCIs) are increasingly present with age in individuals with SCD (2) and are defined as infarcts visible on brain MRI though focal neurological deficits are not apparent (3) SCIs in children with SCD are associated with risk of overt stroke, academic deficits, and further infarction (4, 5), most often occurring in cerebral blood flow (CBF) borderzone regions (6). Thus, 2020 American Society of Hematology guidelines recommend MRI of the brain to screen for SCI at least once in childhood and adulthood (7). SCD also results in cerebral hemodynamic and metabolic aberrations (8). Anemia and altered oxygen affinity of hemoglobin S contribute to low arterial oxygen content (CaO_2_), leading to compensatory increases in CBF (9–11). However, cerebral hemodynamic and metabolic compensation may insufficiently compensate for the extent of anemia (12), thereby leading to subtle brain injury and brain volume loss.

The advent of automated brain segmentation has led to the use of software for the clinical characterization of disorders involving cerebral atrophy such as Alzheimer’s disease (13). Reductions in brain volume may serve as a clinical marker for other diseases such as SCD, however exemplar datasets from generalizable cohorts are not readily available. Furthermore, the relevance of brain volume in SCD has not been conclusively established in the context of tissue infarction and hemodynamic impairment. For instance, prior investigations into SCD and brain volume have reached various conclusions with studies finding reduced gray matter volume (GMV) but not white matter volume (WMV) (14), reduced WMV but not GMV (15) and a longitudinal study that identified decreases in total brain volume over time (16). Studies often were limited by small and heterogenous samples, lower spatial resolution MRI, and variation in field strength and contrast parameters which precludes comparison among data sets. More detailed information on these trends using larger cohorts and contemporary methods could motivate the use of longitudinal brain volume assessment in SCD as an automated marker of disease stability or future progression.

To address this limitation, the purpose of this study was to rigorously evaluate whether children and young adults with SCD, using a larger cohort with more homogenous imaging parameters and standardized inclusion criteria, have reduced GMV and WMV compared to healthy controls using high resolution MRI. We tested the hypotheses that (i) elevated CBF, a marker of cerebral hemodynamic compensation in SCD, is associated with global and regional brain atrophy, and (ii) silent cerebral infarct burden is associated with brain atrophy in excess of infarct volume.

## Materials and methods

This study was approved by the Vanderbilt University Medical Center Institutional Review Board and written informed consent was obtained from all adult participants. For those less than 18 years of age, parents or guardians provided written informed consent and children assented to participate.

**Figure 1:**
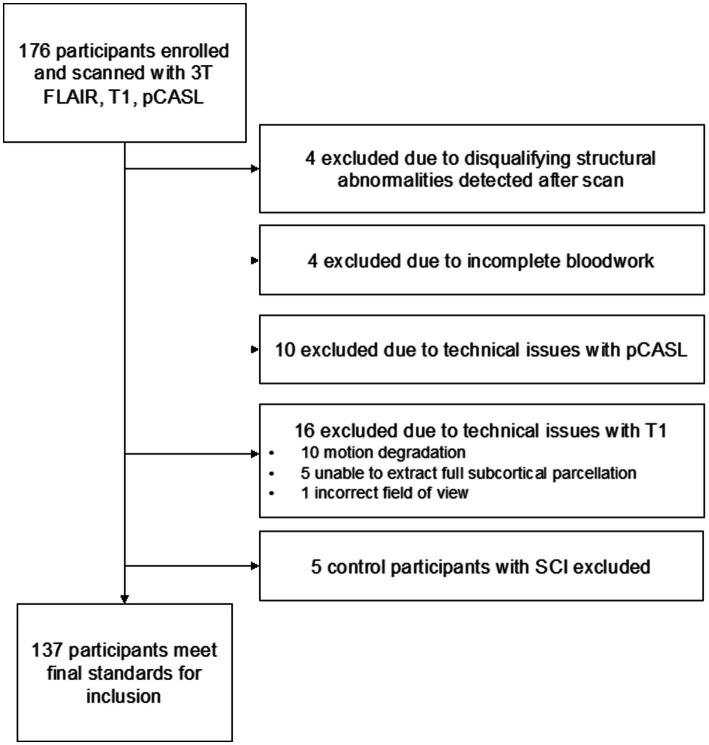
Study exclusion flowchart.

Participants with SCD defined as hemoglobin (Hb) SS or HbSβ0 thalassemia were recruited from SCD clinics at an academic medical center and a community clinic. Race-matched healthy controls (HbAA) were recruited via community outreach; attempts were made to age-match participants within 3 years. SCD inclusion criteria: age = 7–32 years. Exclusion criteria: contraindication to 3 Tesla MRI, prior overt stroke, receiving regular blood transfusions, intracranial stenosis >70%, major neurological or psychiatric condition besides SCD, major structural brain abnormality. Neurological history and exam to confirm that any infarcts were silent, hemoglobin (Hb, g/dL), hematocrit and pulse oximetry (SaO_2,_ %) readings were obtained at time of MRI. Arterial oxygen content (CaO_2_, ml O_2_/100g blood) was calculated as SaO_2_ × Hgb × 1.37. Any SCI in a healthy control participant was an exclusion criterion. Five healthy controls found to have SCIs were excluded from analysis: these five individuals represented a subpopulation too small to adequately analyze but did not otherwise differ from the remaining healthy controls. White matter alterations are expected in a small portion of healthy children and young adults (17, 18). Full exclusion logic is shown in Figure 1.

### Sequences

Each participant was scanned at 3 Tesla (Philips Healthcare, Best, Netherlands). A 3D *T*_1_-weighted magnetization-prepared rapid gradient echo (MPRAGE) sequence (3D turbo-gradient-echo; spatial resolution = 1.0 mm isotropic, TR/TE = 8.20/3.76 ms) was acquired for tissue volume analysis. Axial and coronal 2D fluid-attenuated inversion-recovery (FLAIR) sequences (axial spatial resolution = 0.9 × 1.1 × 3.0 mm; coronal spatial resolution = 0.9 ×  3.0 × 1.1 mm; TR/TI/TE = 11,000/2800/120 ms) were acquired for lesion quantification. 2D pseudocontinuous arterial spin labeling (pCASL) was used to evaluate CBF. Imaging parameters varied slightly between adults and children given expected variations in blood arrival time with age. The labeling volumes were planned so that they were perpendicular to the carotid and vertebrobasilar arteries to reduce the likelihood of the labeling plane traversing the carotid and vertebral arteries obliquely. A common spatial resolution of 3 × 3 × 7 mm and dual-pulse background suppression was used. Adults utilized a pCASL scan with post-labeling delay = 1900 ms (label duration = 1,000 ms), averages = 20, scan duration = 168 s, whereas children utilized an ASL scan with labeling parameters of post-labeling delay = 1,650 ms (label duration = 1,650 ms), averages = 20, scan duration = 168 s. The difference in label delays was due to known differences in blood arrival between adults and children and a desire to titrate each sequence to the population. The shorter label duration was used in the adult protocol as this scan was part of a multi-delay pCASL protocol for transit time determination, and the shorter delay was selected to increase sensitivity to blood arrival time while maintaining adequate signal-to-noise ratio. Here, only the single delay of 1,900 ms was used to increase consistency with the single-delay pediatric scan, and in a direct comparison we have reported that both of these single-delay scans provide CBF values that are reproducible and not significantly different in healthy adults (19). Other scan parameters: TR = 4,200 ms, TE = 13 ms, spatial pre-saturation, bandwidth = 2,665 Hz, slice time = 23 ms, in-plane field-of-view = 240 × 240 mm, slice thickness = 7 mm, slice gap 0.5 mm, slices = 17, SENSE-factor = 2.3, echo planar imaging (EPI) factor = 35, *k*-space trajectory = cartesian. The M_0_ scan was acquired with identical geometry but with the pCASL preparation removed, TR = 20s, and scanner gain, shimming, and scaling unchanged from the pCASL acquisition.

### Image analysis

Tissue segmentations were created using FreeSurfer (version 7.1.1^1^). The standard automated FreeSurfer cortical reconstruction routine (recon-all) was applied to *T*_1_-weighted images; participants with motion corrupted *T*_1_ images were excluded. All resulting segmentations were manually inspected. GNU Parallel was used to reduce computation time (20).

Cerebral blood flow was calculated in each voxel from pCASL acquisitions using the single-PLD kinetic model corrected for the hematocrit, as described previously (21). pCASL data were corrected for motion and baseline drift, normalized by the TR = 20s equilibrium magnetization *M*_0_ scan, and the solution to the flow-modified Bloch equation, using the measured hematocrit for blood *T*_1_ determination and SCD labeling efficiency (*α* = 0.72), was applied (21). The M_0_ image itself was used for normalization, rather than a single measurement in pure blood, as this is now recommended (22), pure arterial blood is not straightforward to isolate, and the image-based approach reduces spatial artifacts from coil sensitivity. Of note, the ISMRM Perfusion Study Group white paper simplified kinetic model (22) was not used; rather, we used an expanded model that accounts for differences in blood and tissue *T*_1_, as well as blood arrival time, which all change in the presence of anemia (23). The primary CBF parameter of interest was the gray matter CBF. For gray matter CBF determination, we utilized the whole brain blood–brain partition coefficient (λ) = 0.9 ml/g, *T*_1,tiss_ = 1.2 s (24, 25), and bolus arrival time (BAT) = 1.10 s for controls and 1.02 s for SCD as measured previously (21). We calculated the participant-specific blood *T_1_* using the measured hematocrit and previously published relationship between arterial (oxygenation = 92 ± 7%) blood water *T_1_* and hematocrit: 1/*T_1_* = 0.52 x Hct + 0.38 (26). An alternative method has been proposed whereby individual *T*_1_ measurements are made in venous blood water *in vivo* (27). We chose not to measure venous *T*_1_ as the flow-modified Bloch equation requires arterial blood water *T*_1_, and the venous and arterial *T*_1_ differ by approximately 200 ms, depending on oxygenation status. However, it should be noted that both approaches are commonly used in the literature (27). Finally, for completeness, we also estimated the white matter CBF as a more exploratory measure. Here, we used the same whole brain blood–brain partition coefficient (λ) = 0.9 ml/g, but estimated BAT at 2.0 s for controls and 1.9 s for SCD under the assumption that transit delays in gray matter and white matter are similar between SCD and healthy participants (28, 29). The resulting CBF maps were registered to the *T_1_*-weighted image using the *M*_0_ image as the template, and the mean CBF within the gray and white matter was separately recorded using the white matter CBF parameters in the white matter mask and gray matter CBF parameters in the gray matter mask.

FreeSurfer estimates whole brain volume and total GMV (30); WMV was calculated as the difference between the total brain volume (excluding ventricles) and GMV. For exploratory analyses, subcortical values were recorded, as well as tissue volumes in all bilateral brain lobes and cerebellum.

Infarcts were traced on axial FLAIR sequences by a staff scientist (RSJ) and independently confirmed by an experienced board-certified neuroradiologist (LTD). For the purposes of this study, only lesions meeting the minimum dimension of a silent cerebral infarct as defined in the Silent Infarct Transfusion Trial (4) (at least 3 mm in one plane and visible in a second imaging plane) were traced. Intracranial volume (ICV) estimates were obtained for each participant; FreeSurfer estimates ICV by dividing a reference atlas ICV by the subject-to-atlas scaling factor calculated during cortical registration.

### Statistical testing

Demographic statistics included mean and standard deviation (normal data) or median and interquartile range (non-normal data) for continuous variables and count and percent for categorical variables. Tests for differences in continuous variables were done with independent samples *t*-tests, or Mann–Whitney *U* tests for non-normally distributed data. Categorial variables were evaluated using a *χ*2 test or Fisher’s exact test for small cell counts. All tests were two-sided.

Multivariate linear regression was used to evaluate the relationship between the presence of SCD as well as physiologic parameters known to be altered by SCD (hemoglobin concentration, CBF, CaO_2_ and lesion burden) and measures of tissue volume. Separate multivariate regressions were created for disease state and each SCD-associated physiologic parameter; these parameters are highly correlated with one another. Separate regressions allows the magnitude of parameter effect on and suitability as a standalone biomarker for changes in brain volume to be quantified and avoids model instability introduced by collinearity.

Models were generated using *statsmodels* (31). All models use age, sex and ICV as covariates in addition to those explicitly stated. Significance was defined as two-sided *p* < 0.05 after Benjamini–Hochberg correction, with each set of regressions using the same independent variable of interest corrected together. Our primary analysis utilized total gray and total white matter volume as dependent variables; a secondary analysis of 66 subcortical and cortical structures was performed as well. Supplementary material includes coefficients for all variables in each regression model are available online.

The infarct burden in our participant population was exponentially distributed; thus, infarct burden was log_10_ transformed before regression. This transformation necessarily removes participants with no lesion burden infarcts from the regression as the logarithm of 0 is undefined.

## Results

A total of 137 participants were included: 88 had SCD (median age = 18.1 years, IQR 15.4; 48.9% male) and 49 were healthy controls (median age = 23.75 years, IQR 12.8; 42.9% male). SCIs were present in 31 participants (35%) with SCD. Participant demographic information is summarized in Table 1.

**Table 1:** Study demographics.

	Control (*n* = 49)	SCD (*n* = 88)	*p* ^a^
Black race, *n* (percent)	49 (100.0%)	86 (97.73%)	0.54^c^
Male sex, *n* (percent)	21 (42.86%)	43 (48.86%)	0.25
Hemoglobin genotype	49 HbAA 100%)	80 HbSS (91%) 8 HbSB0 (9%)	n/a
Age at MRI, years, median (IQR)	23.75 (12.83)	18.06 (15.42)	0.26^d^
SCI present, *n* (percent)	n/a	31 (35.23%)	n/a
SCI count^e^, median (IQR)	n/a	4 (5)	n/a
Total SCI burden^e^, mL, median (IQR)	n/a	0.14 (0.33)	n/a
Gray matter volume, mean (SD)	619.40 (74.16)	626.21 (67.99)	0.588
White matter volume, mean (SD)	441.31 (55.17)	435.62 (62.52)	0.595
GM CBF, ml/100 g/min, mean (SD)	50.64 (8.9)	82.47 (17.92)	<0.001
WM CBF, ml/100 g/min, mean (SD)	24.99 (4.16)	40.49 (10.81)	<0.001
Regular blood transfusions, *n* (percent)	n/a	0 (0.0%)	n/a
CaO_2_, mL/dL, mean (SD)	17.38 (2.12)	11.63 (1.85)	<0.001
SaO_2_, percent, median (IQR)	98.0 (2.0)	96.0 (3.26)	<0.001^d^
Hemoglobin, g/dL, mean (SD)	13.28 (1.57)	9.04 (1.37)	<0.001
Hemoglobin S fraction, percent, median (IQR)	n/a	0.77 (0.16)	n/a
Intracranial stenosis > 70%, *n*, (percent)	0 (0.0%)	0 (0.0%)	n/a
Hydroxyurea therapy, *n* (percent)	n/a	79 (89.77%)	n/a
Type 1 diabetes mellitus^b^, *n* (percent)	3 (6.12%)	2 (2.27%)	0.35^c^
Hypercholesterolemia^b^, *n* (percent)	0 (0.0%)	0 (0.0%)	n/a
Coronary artery disease^b^, *n* (percent)	0 (0.0%)	0 (0.0%)	n/a
Smoking currently, *n* (percent)	2 (4.08%)	8 (9.09%)	0.49^c^
Body mass index, kg/m^2^, mean (SD)	23.29 (8.23)	21.14 (4.54)	0.054

^a^Chi-square test for categorical variables and *t*-test for continuous variables, unless otherwise noted. ^b^Documented in medical records and/or self-reported by study participant. ^c^Fisher exact test used if one cell < 5. ^d^Mann–Whitney *U* test used for non-normally distributed data: interquartile range (IQR) reported instead of SD and median reported instead of mean. ^e^Statistics for lesion count and volume calculated using only participants with 1 or more SCI. CaO_2_, arterial oxygen content; SaO_2_, blood oxygen saturation; SCD, sickle cell disease; SCI, silent cerebral infarct; HbSB0, hemoglobin S β0 thalassemia.

**Figure 2:**
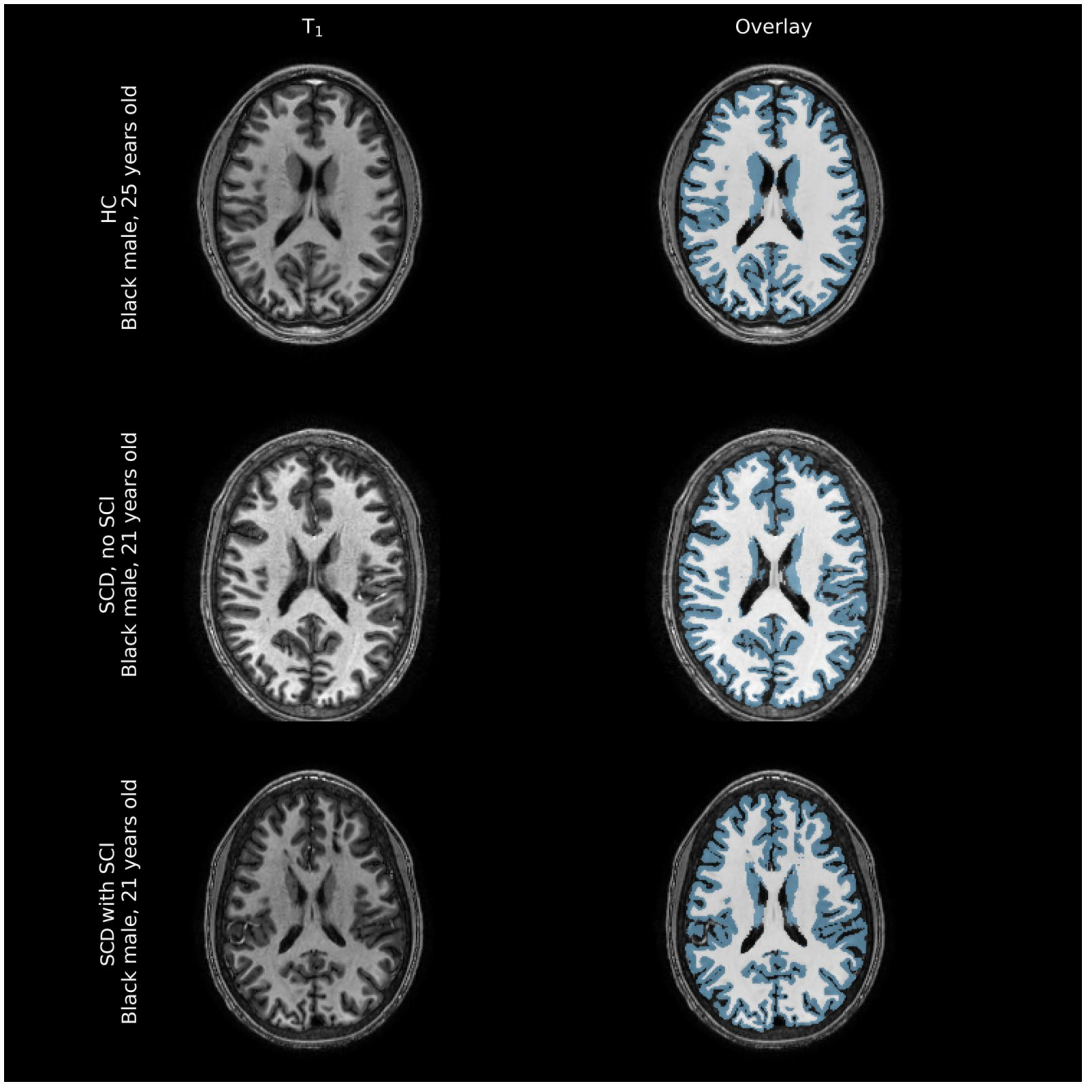
Representative comparison of structural images of participant brains along with FreeSurfer-derived gray and white matter segmentations (blue overlay and white overlay, respectively). Tissue volume differences between healthy controls (HC) and individuals with sickle cell disease (SCD) with and without silent cerebral infarcts (SCI) are often not immediately apparent on structural imaging modalities, barring large overt ischemic strokes that were excluded from this sample.

**Figure 3:**
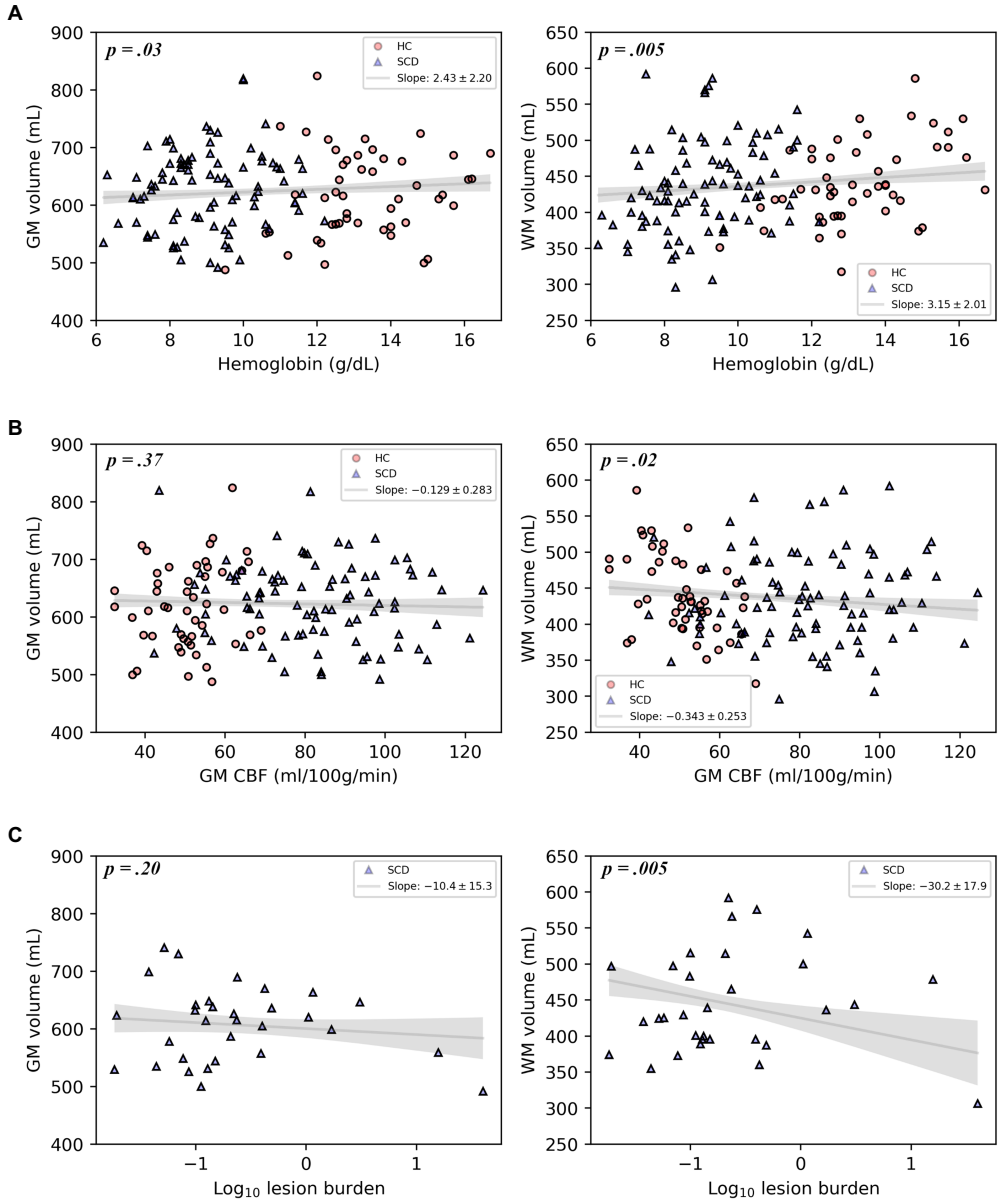
Plots of gray matter volume and white matter volume with hemoglobin, gray matter CBF, or log-transformed infarct (lesion) burden, displaying the multivariable regression model-predicted association between each covariate and volume, controlling for age, sex, and intracranial volume, with 95% confidence intervals (gray line and shaded area). Models include healthy controls and participants with SCD **(A,B)** or participants with SCD and silent cerebral infarcts only **(C)**. Overlaid on the model predictions are the raw data.

Representative comparisons of anatomical images of individuals with and without SCD, along with their FreeSurfer-derived GM and WM segmentations can be seen in Figure 2, demonstrating the quality of the segmentation as well as the minimal differences apparent on standard anatomic neuroimaging in participants with SCD with and without infarcts and healthy controls. Regression plots of total brain volume against factors hypothesized to impact brain size (SCD presence, hemoglobin and infarct burden) are presented in Figure 3.

In the primary multivariable analysis (Table 2) including all study participants, SCD was associated with reduced WMV of 16 mm^3^ after controlling for age, sex and ICV (*p* = 0.0075), compared to healthy controls, Table 2; GMV was not reduced. Lower hemoglobin was associated with decreases in both GMV (*p* = 0.032) and WMV (*p* = 0.005). Reductions in CaO_2_ were associated with reductions in both GMV (*p* = 0.035) and WMV (*p* = 0.006). Increased GM CBF was associated with decreased WMV (*p* = 0.018) but not GMV. In participants with SCD, an increase in infarct burden was associated with diminished WMV (*p* = 0.005). Because infarct burden was log transformed before regressing, the coefficient of this regression (−30.2) translates to approximately 30.2 mm^3^ of WMV loss for each 10-fold increase in infarct burden. Thus, an increase of infarct burden from 0.1 to 1 mm^3^ (the range within approximately two-thirds of infarct burdens for study participants fall) results in an expected WMV deficit that is over 30 times larger than the increase in infarct volume itself. Infarct burden was not associated with GMV.

**Table 2:** Components of multivariable linear regression models for predicting gray and white matter volume (primary analysis), organized by independent variables of interest.

Independent variable	Coefficient	*p*	Dependent variable	Participant group
SCD	−10.4	0.10	GMV	HC + SCD (*n* = 137)
SCD	−15.6	0.008	WMV	HC + SCD (*n* = 137)
Hemoglobin	2.43	0.032	GMV	HC + SCD (*n* = 137)
Hemoglobin	3.15	0.005	WMV	HC + SCD (*n* = 137)
GM CBF	−0.129	0.37	GMV	HC + SCD (*n* = 137)
GM CBF	−0.343	0.018	WMV	HC + SCD (*n* = 137)
CaO_2_	1.76	0.035	GMV	HC + SCD (*n* = 137)
CaO_2_	2.27	0.006	WMV	HC + SCD (*n* = 137)
Infarct burden*	−10.4	0.19	GMV	SCD w/ SCI (*n* = 31)
Infarct burden*	−30.2	0.005	WMV	SCD w/ SCI (*n* = 31)

Each row represents a separate regression model. Corrections for multiple comparisons made using the Benjamini–Hochberg method, treating *p-*values from each independent variable as its own set for correction purposes. All regressions include age, sex and ICV as additional covariates. GM, gray matter; WM, white matter; CBF, cerebral blood flow, HC, healthy control. *Log_10_ of infarct burden.

**Figure 4:**
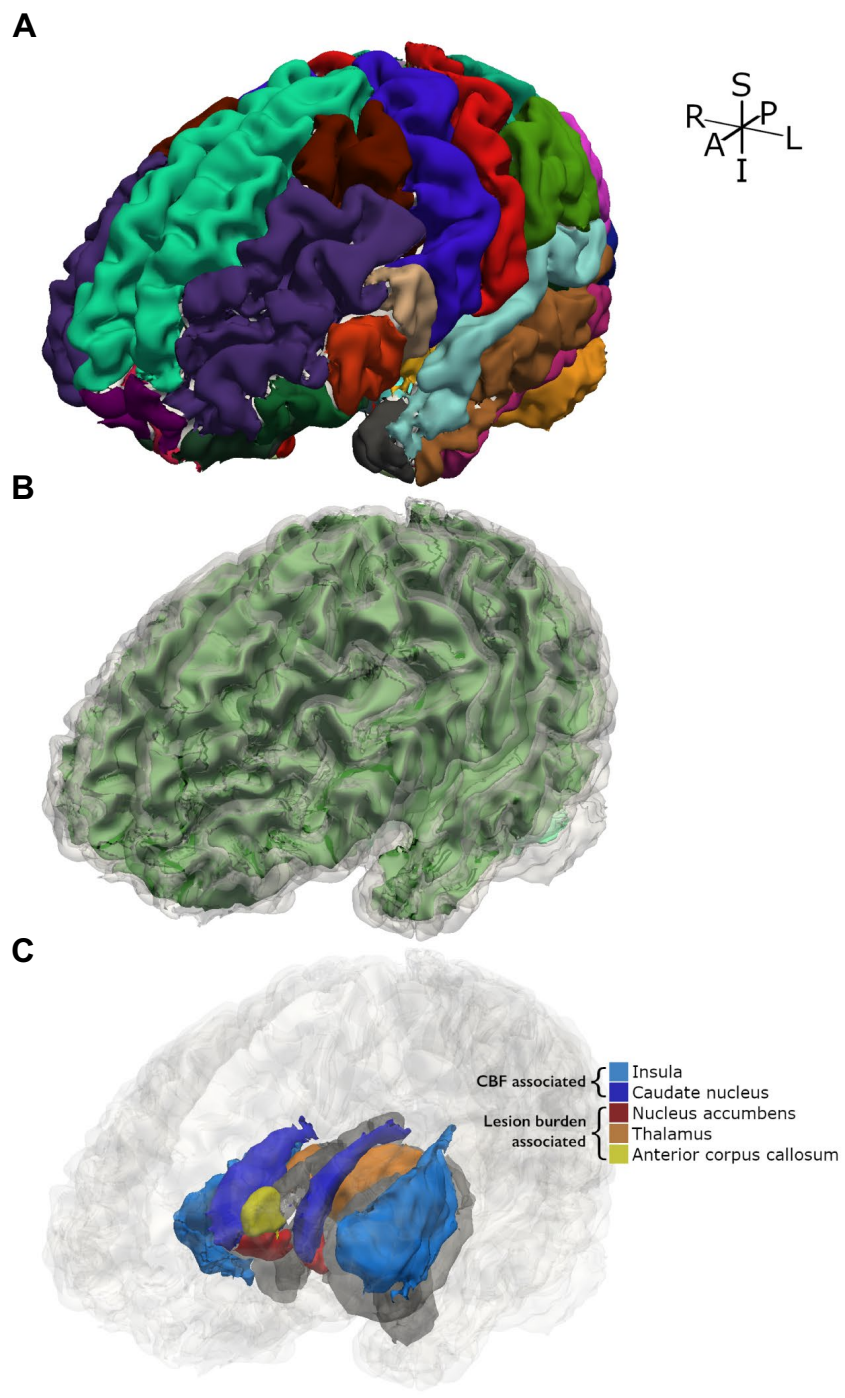
3D parcellation of a representative participant with SCD. **(A)** Shows FreeSurfer cortical parcellation. **(B)** Shows gray matter segmentation (translucent gray) over white matter segmentation (green). **(C)** Shows subcortical and cortical structures for which significant associations with tested independent variables were found. Other structures are displayed in gray as spatial references.

A secondary and exploratory analysis of regional brain volumes is presented in Table 3, which were corrected for multiple volume comparisons as stated in the Methods. Increased GM CBF was associated with decreased volume of the insular lobe (*p* = 0.017), the left caudate nucleus (*p* = 0.017) and the right caudate nucleus (*p* = 0.036). Greater lesion burden was associated with reduced volume of the left nucleus accumbens (*p* = 0.002), the right nucleus accumbens (*p* = 0.002), the right thalamus (*p* = 0.009), and the anterior corpus callosum (*p* = 0.002). Figure 4 shows these structures with reduced brain volume highlighted with a 3-dimesional lobe parcellation. Hemoglobin, CaO_2_ and presence of SCD alone were not associated with any focal deficits in brain volume. Structures with significant associations with CBF or lesion burden are shown in Figure 4.

**Table 3:** Components of multivariable linear regression models for predicting cortical and subcortical structure volumes, organized by independent variables of interest.

Independent variable	Coefficient	*p*	Dependent variable	Participant group
GM CBF	−0.010	0.017	Insula volume	HC + SCD (*n* = 137)
GM CBF	−0.0053	0.017	Left caudate volume	HC + SCD (*n* = 137)
GM CBF	−0.0053	0.036	Right caudate volume	HC + SCD (*n* = 137)
Infarct burden*	−0.11	0.002	Left nucleus accumbens volume	SCD w/ SCI (*n* = 31)
Infarct burden*	−0.085	0.002	Right nucleus accumbens volume	SCD w/ SCI (*n* = 31)
Infarct burden*	−0.44	0.009	Right thalamus volume	SCD w/ SCI (*n* = 31)
Infarct burden*	−0.098	0.002	Anterior corpus callosum volume	SCD w/ SCI (*n* = 31)

Each row represents a separate regression model. Due to space constraints, only significant models are shown. Corrections for multiple comparisons made using the Benjamini–Hochberg method, treating the *p-*values from each independent variable as its own set for correction purposes. All regressions include age, sex and ICV as additional covariates. GM, gray matter; WM, white matter; CBF, cerebral blood flow. *Log_10_ of infarct burden.

## Discussion

In a relatively well-characterized cohort of children and young adults with SCD treated at a single institution and excluding potential confounding comorbidities (e.g., overt stroke, regular blood transfusion therapy, vasculopathy), we found SCD to be associated with decreased WMV compared to race and sex matched controls (total *N* = 137). The measured brain volume deficits, however, were subtle: SCD was associated with a 16 mm^3^ loss of WMV after controlling for age, sex, and ICV. The small magnitude of the volume (32) difference hints at the complexity of volumetric brain analysis of individuals with SCD and may explain the variable findings in prior studies of volumetric deficits related to SCD.

Interestingly, WMV losses associated with silent infarction in our study outpaced the measured infarct volume by a factor of 30; the WMV deficits seen are too large to be explained by loss in tissue volume at the site of infarction alone. This volume loss suggests that global white matter injury cannot be easily visualized with typical clinical imaging modalities may co-occur with silent infarction. One possible explanation supported by prior work is that white matter microstructural injury occurs prior to and possibly concurrently with the development of cerebral infarcts in SCD, which has been visualized with diffusion tensor imaging (33); our cohort does not have diffusion tensor data for evaluation. Furthermore, increased infarct burden is associated with focal volume loss in several subcortical structures including bilateral volume loss in the nucleus accumbens, a structure partly involved in learning, executive function, and impulse control (34). Increases in infarct burden were also associated with decreased thalamic volume, though the effect was only significant in the right thalamus. Given that there is no biological evidence otherwise to suggest why volumetric losses would be unilateral, we expect that the true effect is bilateral. Though SCI has been defined as infarction without focal neurological deficits, subcortical volume deficits associated with SCI may partly explain why individuals with SCD and SCI have a high risk of executive and cognitive dysfunction (5, 15).

Reductions in hemoglobin and CaO_2_ were both associated with deficits in GMV and WMV. These findings extend the work of a previous study that showed reductions in hemoglobin were associated with WMV deficits (15). However, neither hemoglobin nor CaO_2_ were associated with focal changes in brain volume whereas GM CBF was associated with bilateral losses of volume in the caudate nuclei, structures important for stimulus control learning (35), as well as the insular cortex. Overall, brain volume losses were associated with elevated CBF, consistent with impaired cerebral hemodynamic compensation in SCD (10).

This work adds to the SCD literature, where prior studies have come to various conclusions about brain volumes in people with SCD. One previous study, using a semi-automated neural network to classify brain tissue, reported gray matter development was delayed in 83 children with SCD compared to 43 healthy controls (14), while another found decreased hemoglobin was associated with reduced WMV in a cohort of 52 adults and children with SCD, 26 with non-sickle cell anemia and 40 controls (15) when using BrainSuite’s tensor-based morphometry.^2^ Using FSL-FAST, a decrease in total brain volume in 34 adults with SCD compared to 11 controls was found (11); these participants were included in the cohort presented in this study. A clinical trial investigating monthly blood transfusions for the prevention of new silent infarcts in 196 children with SCD and SCI which utilized SIENA and paired MRIs at study entry and exit suggested that SCD results in whole-brain volume loss over time (16). These studies excluded patients with overt stroke but are complicated by variable MRI resolution (1.5 versus 3 T), heterogeneous, intermediate-sized cohorts and the complexities of SCI, vasculopathy, age, and different software, possibly accounting for the variability in conclusions regarding the relationship between SCD and brain volume. The breadth of methods and consistent prior findings of reductions in some aspect of brain volume suggests a real effect, but the subtlety of the deficits highlights the relevance of functional indicators of hemodynamic compensation in SCD (6, 11, 36) and longitudinal follow up.

Our study differs from previous cross-sectional investigations of brain volume in SCD with reasonably large cohort that includes both children and adults and includes assessment of CBF in the analysis of brain volume. We excluded SCD participants undergoing regular blood transfusions because blood assays were collected at one time point and may not be representative of physiological steady state; hemoglobin and oxygen saturation levels peak after transfusion and then steadily decrease. Individuals receiving transfusions represent a more clinically symptomatic subset of individuals with SCD compared to those without, so we expect this group would exhibit the greatest brain volume deficits. Thus, our cohort is a healthier group of participants with SCD. Our findings may not be representative of deficits in cohorts with overt stroke, severe cerebral vasculopathy, and those receiving aggressive therapies, for whom we would expect even more severe brain volumetric deficits.

Study strengths include a larger sample size and race-matched healthy controls than evaluated in previous cross-sectional studies of brain volume in SCD with a larger range in ages, along with 3 Tesla high resolution MRI for all participants. This is also the first study to examine either CBF or a continuous measure of infarct burden volume as a marker of brain volume in SCD. Limitations of our study include cross-sectional design, which poses issues for detecting subtle effects or the ordering of associations, but our cohort of 137 participants provides good sensitivity for a study of this design and our limited selection of covariates used in our models limits overfitting.

Brain volumes have been measured in SCD clinical trials and have been used clinically in other conditions such as Alzheimer’s disease. The findings presented in this work suggest that common physiological consequences of SCD (reduced hemoglobin, reduced CaO_2_, increased GM CBF, increased risk of silent infarction) and SCD itself result in both diffuse and focal brain tissue volume deficits. Still, these deficits are subtle, and so brain volume measurements at a single timepoint should be treated cautiously. Hemodynamic and metabolic changes, approximately 1.5-fold different in SCD vs. healthy race-matched controls, are stronger biomarkers of disease severity though they require more advanced neuroimaging techniques (9, 37). Additionally, the white matter volume deficits associated with silent infarction are more than 30 times larger than the infarct volume itself. A possible explanation is that a common mechanism controls infarction risk and volume loss, and that the presence of infarcts in SCD could be a visible marker of subtle diffuse brain injury. The consistent reduction in WMV is logical because despite elevated CBF, silent infarcts primarily occur in borderzone of cerebral blood flow which is predominantly subcortical white matter. Vaclavu et al. (37) also found CBF was higher in patients with SCD versus controls; after an acetazolamide challenge causing cerebral vasodilation and further elevating CBF, cerebral oxygen utilization seemed to worsen in SCD patients. Juttukonda et al. (38) and others (39) have proposed that elevated CBF may lead to capillary level shunting. This work suggests that elevated CBF may not be nourishing to the brain, indicating a mechanism other than cerebral infarction that may lead to brain volume loss.

While the current study is cross-sectional, further longitudinal investigation of the relationships between SCI, brain volume losses and cognitive impairment in SCD may enable the use of infarct burden and brain volume assessments as easily accessible and potentially automated prognostic markers of impairment that could then guide clinical care decisions. This is currently clinically relevant because in many settings, MRI of the brain will be completed as part of standard care for SCD at least once in childhood and once in early adulthood. To allow longitudinal assessment of brain volume from clinically available neuroimaging, we recommend that MRI of the brain be acquired at the same field strength, ideally 3 T, and with a 3D-T1 imaging sequence that will allow brain volume assessment over time. The associations of infarct burden and CBF with volumetric deficits in specific structures suggest potential as biomarkers of specific structure-related impairment, which future studies may elucidate.

## Data availability statement

The raw data supporting the conclusions of this article will be made available by the authors, without undue reservation.

## Ethics statement

The studies involving human participants were reviewed and approved by Vanderbilt University Medical Center Institutional Review Board. Written informed consent to participate in this study was provided by the participants’ or for those less than 18 years of age, by legal guardian. Children also provided assent.

## Author contributions

RJ: study conception and design, data acquisition, analysis and interpretation of data, and manuscript drafting and revision. SW: data acquisition, analysis and interpretation of data, and manuscript revision. LD, AK, SP, and MRD: interpretation of data and manuscript revision. MR: analysis and interpretation of data and manuscript revision. CC and NP: data acquisition and manuscript revision. MJD and LJ: study conception and design, analysis and interpretation of data, and manuscript revision. All authors contributed to the article and approved the submitted version.

## Funding

The authors acknowledge receipt of the following financial support for the research, authorship, and/or publication of this article: the NIH (K24-HL147017, R01 NS096127, R01 NS097763, and UL1 TR000445) and the American Heart Association (AHA #14CSA20380466).

## Conflict of interest

MJD is a paid consultant for Global Blood Therapeutics, receives advisory board receives research-related support from Philips North America, and is the CEO of Biosight LLC, which provides healthcare technology consulting services. These agreements have been approved by Vanderbilt University Medical Center in accordance with its conflict-of-interest policy. MR is an independent statistical consultant. He owns Rodeghier Consulting, Chicago, IL, United States. He was paid for his work on this manuscript via NIH grant funding. MRD and his institution are the sponsor of two externally funded research investigator-initiated projects. Global Blood Therapeutics will provide funding for the cost of these clinical studies but will not be a cosponsor of either study. MRD is not receiving any compensation for the conduct of these two-investigator initiated observational studies. MRD is a member of the Global Blood Therapeutics advisory board for a proposed randomized controlled trial for which he receives compensation. MRD is the steering committee for a Novartis-sponsored phase 2 trial to prevent priapism in men. MRD was a medical advisor for the development of the CTX001 Early Economic Model. MRD provided medical input on the economic model as part of an expert reference group for Vertex/CRISPR CTX001 Early Economic Model in 2020. MRD provided a onetime consultation to the Forma Pharmaceutical company about sickle cell disease in 2021.

The remaining authors declare that the research was conducted in the absence of any commercial or financial relationships that could be construed as a potential conflict of interest.

## Publisher’s note

All claims expressed in this article are solely those of the authors and do not necessarily represent those of their affiliated organizations, or those of the publisher, the editors and the reviewers. Any product that may be evaluated in this article, or claim that may be made by its manufacturer, is not guaranteed or endorsed by the publisher.

## Footnotes

^1^ http://surfer.nmr.mgh.harvard.edu

^2^ http://brainsuite.org/
